# N-Terminal Fatty Acids of NEF^MUT^ Are Required for the CD8^+^ T-Cell Immunogenicity of In Vivo Engineered Extracellular Vesicles

**DOI:** 10.3390/vaccines8020243

**Published:** 2020-05-22

**Authors:** Chiara Chiozzini, Francesco Manfredi, Claudia Arenaccio, Flavia Ferrantelli, Patrizia Leone, Maurizio Federico

**Affiliations:** National Center for Global Health, Istituto Superiore di Sanità, Viale Regina Elena, 299, 00161 Rome, Italy; chiara.chiozzini@iss.it (C.C.); francesco.manfredi@iss.it (F.M.); claudia.arenaccio@iss.it (C.A.); flavia.ferrantelli@iss.it (F.F.); patrizia.leone@iss.it (P.L.)

**Keywords:** CD8^+^ T-cell immunity, exosomes, extracellular vesicles, HIV-1 Nef, DNA immunization

## Abstract

We recently described a cytotoxic CD8^+^ T lymphocyte (CTL) vaccine platform based on the intramuscular (i.m.) injection of DNA eukaryotic vectors expressing antigens of interest fused at the C-terminus of HIV-1 Nef^mut^, i.e., a functionally defective mutant that is incorporated at quite high levels into exosomes/extracellular vesicles (EVs). This system has been proven to elicit strong CTL immunity against a plethora of both viral and tumor antigens, as well as inhibit both transplantable and orthotopic tumors in mice. However, a number of open issues remain regarding the underlying mechanism. Here we provide evidence that hindering the uploading into EVs of Nef^mut^-derived products by removing the Nef^mut^ N-terminal fatty acids leads to a dramatic reduction of the downstream antigen-specific CD8^+^ T-cell activation after i.m. injection of DNA vectors in mice. This result formally demonstrates that the generation of engineered EVs is part of the mechanism underlying the in vivo induced CD8^+^ T-cell immunogenicity. Gaining new insights on the EV-based vaccine platform can be relevant in view of its possible translation into the clinic to counteract both chronic and acute infections as well as tumors.

## 1. Introduction

The immune system protects from both external and internal health threats. In many cases, when the natural immune responses cannot contain the pathology, the intake of immunogenic compounds can block the pathogenic process. This typically occurs in the vaccine-dependent induction of neutralizing antibodies against several infective pathogens. Differently, the identification, production, and marketing of CD8^+^ T-cell-based vaccines are much more restricted, although there is a wide consensus about their potential usefulness against infectious and tumor diseases [1,2].

All cell types spontaneously release nanovesicles, which depending on the respective biogenesis, can be identified as exosomes and microvesicles/ectosomes [3]. Exosomes are vesicles of 50–150 nanometers (nm) which form intracellularly upon inward invagination of endosome membranes. Emerging intraluminal vesicles (ILVs) then accumulate into multivesicular bodies (MVBs), which can traffic either to lysosomes for degradation or to plasma membrane before release. Nanovesicles having both physical and biochemical features resembling exosomes but generated through the direct extrusion of the plasma membrane and with a diameter up to 500–1000 nm are referred to as ectosomes/microvesicles. Together, these nanovesicles are defined as extracellular vesicles (EVs).

We developed an original vaccine platform based on intramuscular (i.m.) injection of DNA eukaryotic vectors expressing a Human Immunodeficiency Virus (HIV)-1 Nef mutant (i.e., Nef^mut^) devoid of the typical biologic activities of wt Nef, but incorporating into EVs at quite high levels even when fused at its C-terminus with foreign antigens [4]. The i.m. injection of a DNA vector expressing any antigen of interest fused to Nef^mut^ induced a strong antigen-specific CD8^+^ T-cell immune response [5]. Moreover, when Nef^mut^ was fused with the viral tumor-specific antigen HPV16-E7, the immune response was strong enough to inhibit the growth of transplantable E7-expressing tumors [6]. Similarly, the i.m. injection of a DNA vector expressing the product of the fusion between Nef^mut^ and the extracellular domain of HER2/Neu generated a strong inhibition of orthotopic tumors in MMTV-HER2/neu transgenic mice [7]. Taken together, these results demonstrated that the Nef^mut^-based vaccine platform could have widespread applications. For these reasons, investigating the mechanism underlying the induction of CD8^+^ T-cell immunogenicity in vivo is relevant. In an effort towards this direction, we here demonstrate that the i.m. injection in mice of a DNA vector expressing a Nef^mut^ isoform unable to be uploaded into exosomes/EVs failed to induce the antigen-specific CD8^+^ T-cell immune response. Thus, the Nef^mut^ uploading in exosomes/EVs is essential for the induction of CD8^+^ T-cell immunity. This result represents a significant achievement towards a complete clarification of the mechanism on the basis of the antigen-specific CD8^+^ T-cell activation-induced in vivo by i.m. injection of DNA vectors expressing Nef^mut^ derivatives.

## 2. Materials and Methods

### 2.1. DNA Constructs

**Figure 1:**
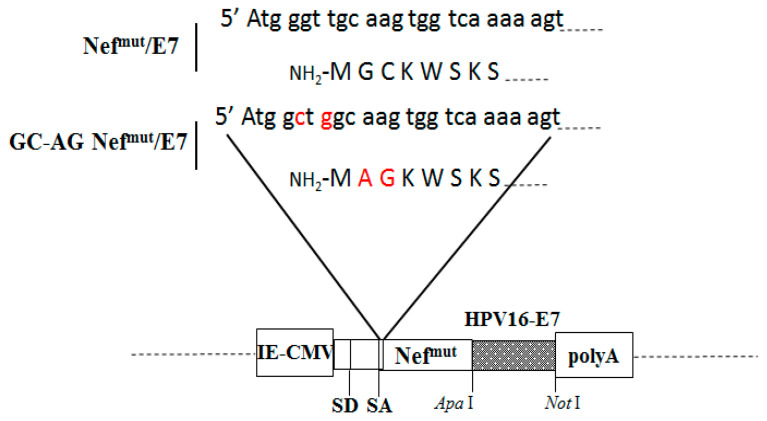
Structure of the DNA molecular constructs expressing Nef^mut^-based products. On the top, shown are both nucleotide and amino acid N-terminal sequences of both Nef^mut^/E7 and GC-AG Nef^mut^/E7. Mutated sequences of the latter are highlighted in red. At the bottom, the structure of both DNA vectors is reported, including the restriction sites where the HPV16-E7 open reading frame was inserted. IE-CMV: immediate-early CMV promoter; SD: major splice donor site; SA major splice acceptor site; polyA: polyadenylation site.

The expression of all open-reading frames (ORFs) were regulated by an immediate-early cytomegalovirus (IE-CMV) promoter in the context of pTargeT vectors (Invitrogen). The GC-AG Nef^mut^/E7 vector was constructed starting from PCR-amplification of the Nef^mut^/E7 vector [8,9] using a forward primer including the unique Mlu I restriction site as well as the GC to AG mutation (Figure 1), and a reverse oligo carrying the unique Apa I site at the end of Nef^mut^ ORF. The amplified DNA fragment was then double digested by Mlu I/Apa I restriction enzymes, and then ligated to the Nef^mut^/E7 construct after deletion of the Nef^mut^ ORF by Mlu I/Apa I digestion. The resulting fusion sequence was finally checked by sequencing for the presence of unwanted nucleotide mutations/deletions. The Nef^mut^ DNA expressing vector was already described [4].

### 2.2. Cell Cultures and Transfection

Human embryonic kidney (HEK)-293T cells (ATCC, CRL-11268) were grown in DMEM (Gibco) plus 10% heat-inactivated fetal calf serum (FCS). Transfection assays were performed using Lipofectamine 2000 (Invitrogen, Thermo Fisher Scientific, Waltham, MA, USA). Cycloheximide (Sigma, St. Louis, MO, USA) was used at a final concentration of 1 μg/mL. 

For the ex vivo immunogenicity test, spleens recovered from sacrificed mice were transferred into a 60-mm Petri dish containing 2 mL of RPMI 1640 (Gibco, Thermofisher Scientific, Loughborough, UK), 50 µM 2-mercaptoethanol (Sigma, St. Louis, MO, USA). Splenocytes were extracted by incising the spleen with sterile scissors and pressing the cells out of the spleen sac with the plunger seal of a 1-mL syringe. After the addition of 2 mL of RPMI medium, cells were transferred into a 15 mL conical tube and the Petri plate was washed with 4 mL of medium to collect the remaining cells. After three-minute sedimentation, splenocytes were transferred to a new sterile tube to remove cell/tissue debris. Live cells were counted by the trypan blue exclusion method.

### 2.3. Exosome Purification

Cells transfected with vectors expressing the Nef^mut^-based fusion proteins were washed 24 h later and reseeded in complete medium. The supernatants were harvested from 48 to 72 h after transfection. Exosomes were recovered through differential centrifugations [10,11] by centrifuging supernatants at 500× *g* for 10 min, and then at 10,000× *g* for 30 min. Supernatants were harvested, filtered with 0.22-μm pore size filters, and ultracentrifuged at 70,000× *g* for l h. Pelleted vesicles were resuspended in 1 × PBS, and ultracentrifuged again at 70,000× *g* for 1 h. Afterwards, pellets containing exosomes were resuspended in 1:100 of the initial volume.

### 2.4. Confocal Microscope Analysis

A total of 4 × 10^4^ HEK-293T cells was seeded on chamber glass slides (BD Biosciences, San Diego, CA, USA) and transfected with vectors expressing either Nef^mut^, Nef^mut^/E7, or GC-AG Nef^mut^/E7. Forty-eight hours later, cells were permeabilized through the Cytofix-Cytoperm-based protocol (BD Biosciences, San Diego, CA, USA) and then labeled with 1:2000 diluted anti-Nef mAb MATG020 (kindly provided by O. Schwartz, Paris, France), followed by incubation with 1:2500 diluted Alexa 488-conjugated goat anti-mouse (Invitrogen). Coverslips were mounted using an anti-fade mounting medium containing 4’-6-diamidino-2-phenylindole (DAPI). Images were acquired using a Leica TCS SP5 confocal microscope and analyzed by the LAS AF version 1.6.3 software (Leica microsystems, Wetzlar, Germany).

### 2.5. Western Blot

Western blot analyses of both cell lysates and exosomes were carried out as described [4] after resolving samples in 10% sodium dodecyl sulfate-polyacrylamide gel electrophoresis (SDS-PAGE). In brief, Western blot analysis on cell lysates was performed by washing cells twice with 1 × PBS (pH 7.4) and lysing them with 1 × SDS-PAGE sample buffer. Samples were resolved by SDS-PAGE and transferred by electroblotting on a 0.45-μM pore size nitrocellulose membrane (Amersham) overnight using a Bio-Rad (Hercules, CA, USA) Trans-Blot. For Western blot analysis of exosomes, they were lysed and analyzed as described for cell lysates. For immunoassays, membranes were blocked with 5% non-fat dry milk in PBS containing 0.1% Triton X-100 for 1 h at room temperature, then incubated overnight at 4 °C with specific antibodies diluted in PBS containing 0.1% Triton X-100. Filters were revealed using 1:1000-diluted sheep anti-Nef antiserum ARP 444 (MHRC, London, UK), 1:500-diluted anti-β-actin AC-74 mAb from Sigma (St. Louis, MO, USA), and 1:500 diluted anti-Alix H-270 polyclonal Abs from Santa Cruz (Heidelberg, Germany). Densitometry analysis was carried out with Bio-Rad Image Lab software of a ChemiDoc imager.

### 2.6. Mice Immunization and IFN-γ ELISpot Assay

The studies with animals were approved by the Italian Ministry of Health, authorization *n*. 950/2018, according to Legislative Decree 116/92, which was implemented in Italy by the European Directive 86/609/EEC on laboratory animal protection. Animals used in our research were purchased from Charles River Laboratories Italia (Calco, Italy), and housed and treated according to the guidelines inserted in the above-mentioned Legislative Decree. A total of eight mice for each experiment were inoculated i.m. two times in fourteen-day intervals with 50 μg for each quadriceps of DNA vectors purified through endotoxin-free Qiagen kit (Hilden, Germany). Fifteen days after the last inoculation, mice were sacrificed and splenocytes isolated from spleens. For the IFN-γ ELISpot assay, 2.5 × 10^5^ live cells were seeded in each microwell. Cultures were run in triplicate in ELISpot multiwell plates (Millipore, cat *n*. MSPS4510) pre-coated with the AN18 mAb against mouse IFN-γ (Mabtech, Nacka Strand, Sweden) in RPMI 1640 plus 10% FBS for 16 h in the presence of 5 µg/mL of the HPV16-E7-specific nonamers 21–28, DLYCYEQL, and 49–57, RAHYNIVTF [12]. As a negative control, 5 µg/mL of the H2-K^b^-binding HCV-NS3 specific peptide ITQMYTNV [13] were used. For cell activation control, cultures were treated with 10 ng/mL PMA (Sigma St. Louis, MO, USA) plus 500 ng/mL of ionomycin (Sigma, St. Louis, MO, USA). After 16 h, cultures were removed, and the wells incubated with 100 µL of 1 µg/mL of the R4–6A2 biotinylated anti-IFN-γ (Mabtech, Nacka Strand, Sweden) for 2 h at r.t. Wells were then washed and treated for 1 h at r.t. with 1:1000 diluted streptavidine-ALP preparations from Mabtech. After washing, spots were developed by adding 100 µL/well of SigmaFast BCIP/NBT. The spot-forming cells were finally analyzed and counted using an AELVIS ELISpot reader.

### 2.7. Statistical Analysis

When appropriate, data are presented as mean values + standard deviation (SD).

## 3. Results

### 3.1. The Lack of Fatty Acids at the Nef^mut^/E7 N-Terminus Affects the Intracellular Localization but Not the Protein Stability 

The actual role of in vivo engineered EVs in the induction of antigen-specific CD8^+^ T-cell immunity after i.m. injection of Nef^mut^-based DNA vectors [5,6,7] represented a still unmet, but crucial issue. We assumed that finding a direct correlation between exosome uploading and CD8^+^ T-cell immunogenicity in the context of different Nef^mut^ isoforms would adequately address the issue. To optimize the sensitivity of downstream immunogenicity assays in mice, we used a Nef^mut^-derivative fused at its C-terminus with HPV16-E7 in view of the already proven strong immunogenicity of E7 in this configuration [6].

To identify a Nef^mut^ isoform with an impaired ability to associate with exosomes, we hypothesized that hindering the interaction with cell membranes would decrease its efficiency of uploading in exosomes. Therefore, we recovered a Nef^mut^/E7 isoform (here referred to as GC-AG Nef^mut^/E7) lacking the signals for both myristoylation and palmitoylation at its N-terminus (Figure 1), i.e., post-translation modifications driving the Nef interaction with cell membranes [14].

**Figure 2:**
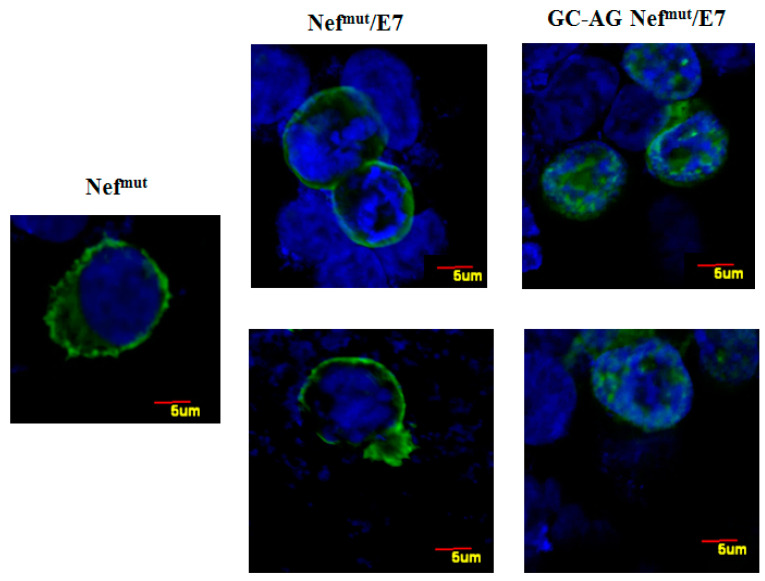
Intracellular localization of both Nef^mut^/E7 and GC-AG Nef^mut^/E7. Confocal microscope analysis of HEK-293T cells transfected with either Nef^mut^, Nef^mut^/E7, or GC-AG Nef^mut^/E7 expressing vectors. Shown are representative fields from transfected cell cultures incubated first with an anti-Nef mAb, and then with Alexa 488-conjugated anti-mouse IgGs. DAPI (blue fluorescence) was used to highlight cell nuclei. Scale bars are reported. The results are representative of two independent experiments.

In the first instance, the intracellular localization of GC-AG Nef^mut^/E7 was compared to that of the parental isoform by confocal microscope analysis (Figure 2). We noticed that, whereas parental Nef^mut^/E7 mostly localized, as previously described [15], at the inner leaflet of the plasma membrane, the GC-AG mutant mainly accumulated into the nucleus, likely consequence of the natural intracellular localization of E7 [16]. This evidence is consistent with the idea that the N-terminal fatty acids are important for the membrane association of Nef^mut^/E7.

**Figure 3:**
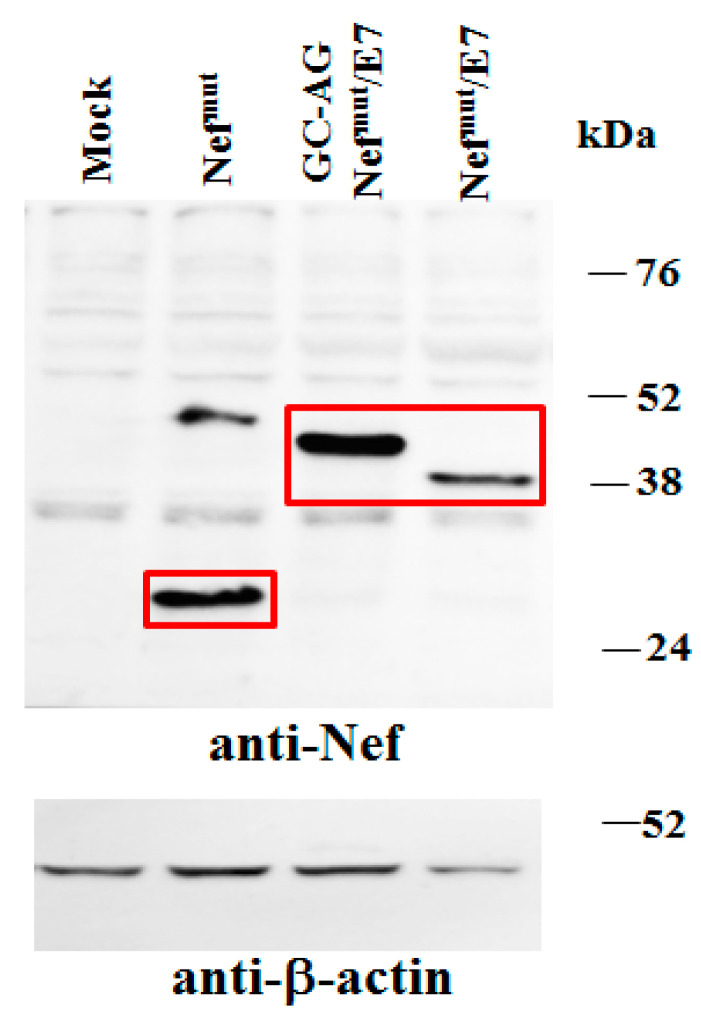
Analysis of expression of GC-AG Nef^mut^/E7. Western blot analysis of total cell lysates from HEK-293T cells transfected with DNA vectors expressing the indicated Nef^mut^-based fusion products. As control, conditions from mock-transfected cells, as well as cells transfected with Nef^mut^, were included. Polyclonal anti-Nef Abs served to detect Nef^mut^-based products, while β-actin levels were detected as a reference standard. Nef-specific signals are highlighted. Molecular markers are given in kilodaltons (kDa). Signal intensity ratios compared to the Nef^mut^ control condition, considering the respective β-actin signals, were 1.48 and 1.43 for GC-AG Nef^mut^/E7 and Nef^mut^/E7, respectively. The results are representative of five independent experiments. Uncropped blots showing all the bands with all molecular weight markers are shown in Supplementary Figure S1.

Next, to assess whether the different intracellular localization of GC-AG Nef^mut^/E7 affects its stability, comparative analyses with the parental fusion protein were carried out. In particular, steady-state levels of the two products were first compared by transfecting 5 × 10^4^ HEK-293T cells in microwells with vectors expressing each Nef^mut^-derivative and, as control, Nef^mut^ alone. After 48 h, cells were washed, lysed, and half of the total cell lysates were analyzed by Western blot. Considering the respective β-actin signals, no major differences between steady-state levels of Nef^mut^/E7 and GC-AG Nef^mut^/E7 were noticed (Figure 3). Notably, in our hands, Nef molecules deprived of their N-terminal fatty acids reproducibly migrated a bit slower compared to parental isoforms, likely due to differences in mass-to-charge ratios.

**Figure 4:**
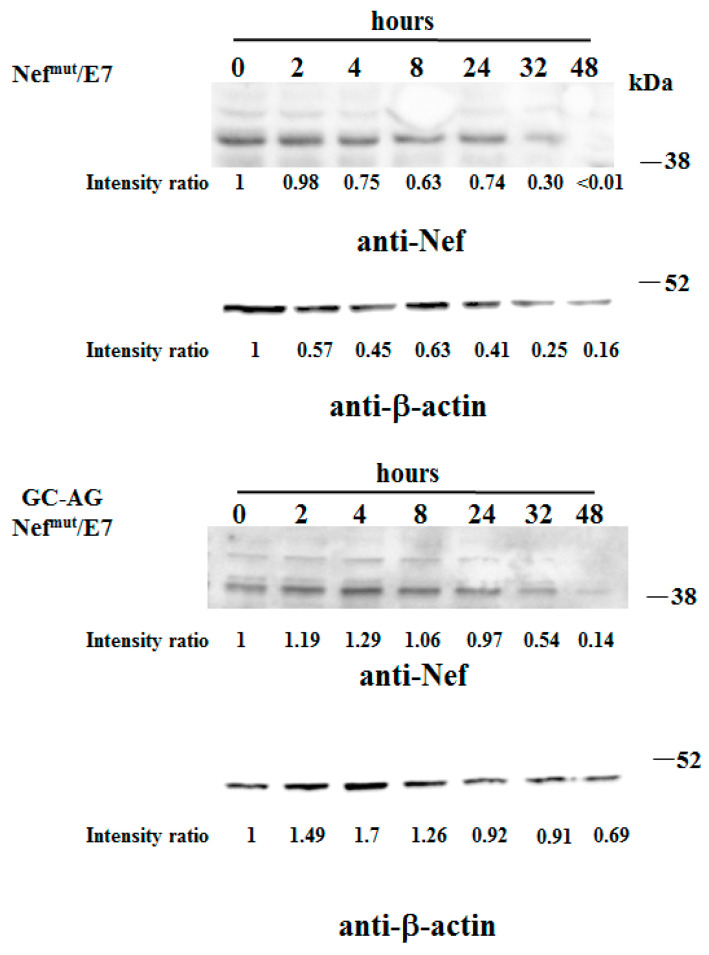
Analysis of stability of GC-AG Nef^mut^/E7. Comparative stability analysis between Nef^mut^/E7 and GC-AG Nef^mut^/E7 fusion proteins in total lysates from HEK-293T cells transfected with the respective DNA vectors. Forty-eight hours after transfection, cells were washed and reseeded in complete medium containing 1 μg/mL of cycloheximide. Transfected cells were harvested at the indicated times, and total lysates from the same number of cells were analyzed by Western blot assay. Filters were revealed by both anti-Nef and anti- β-actin polyclonal antibodies. Time zero refers to cells harvested at the time of cycloheximide treatment. Signal intensity ratios compared to respectve time zero are indicated. Molecular markers are given in kDa. The results are representative of two independent experiments. Uncropped blots showing all the bands with all molecular weight markers are shown in Supplementary Figure S2.

The intracellular stability of the two fusion products was assessed by treating cells with cycloheximide to halt their synthesis after DNA transfection, and evaluating the amounts of the two products over time by Western blot analysis. We observed the disappearance of both Nef^mut^/E7 and GC-AG Nef^mut^/E7 signals 48 h after cycloheximide treatment (Figure 4), indicating that the kinetics of intracellular degradation of the two fusion products was similar. This result supports the idea that the lack of N-terminal fatty acids does not affect the overall stability of Nef^mut^/E7.

### 3.2. N-Terminal Fatty Acids Are Required for Exosome Incorporation of Nef^mut^/E7

**Figure 5:**
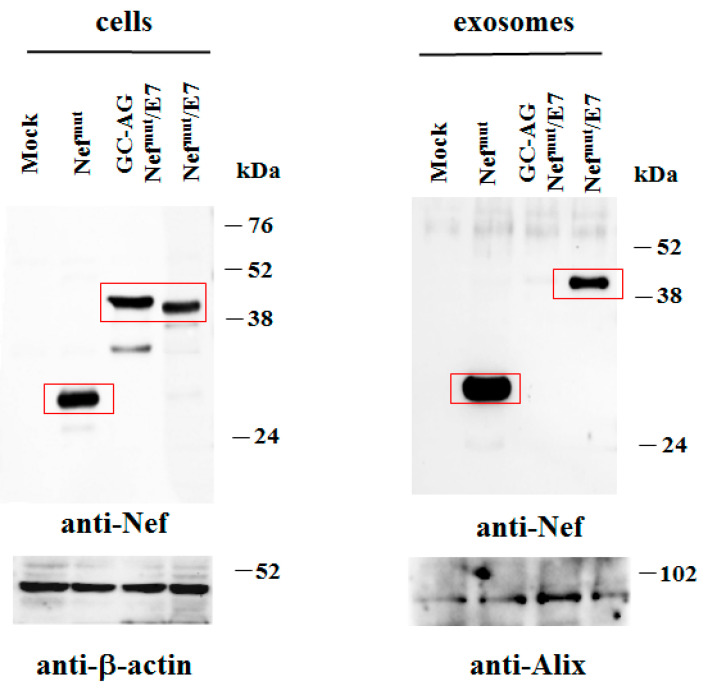
Detection of Nef^mut^/E7 based fusion products in transfected cells and exosomes. Shown is the Western blot analysis of total lysates from the same number of HEK293T cells transfected with DNA vectors expressing either Nef^mut^/E7 or GC-AG Nef^mut^/E7 (left panels). Equal volumes of buffer, where purified exosomes were resuspended after differential centrifugations of the respective supernatants, were also analyzed (right panel). As control, conditions from mock-transfected cells, as well as cells transfected with Nef^mut^ alone, were included. Polyclonal anti-Nef Abs served to detect Nef^mut^-based products, while β-actin and Alix were revealed as markers for cell lysates and exosomes, respectively. Concerning signals from cell lysates, intensity ratios compared to the Nef^mut^ control condition, considering the respective β-actin signals, were 1.05 and 0.91 for GC-AG Nef^mut^/E7 and Nef^mut^/E7, respectively. Regarding exosomes, the intensity ratio between Nef^mut^ and Nef^mut^/E7 conditions, considering the respective Alix signals, was 0.81. Molecular markers are given in kDa. The results are representative of five independent experiments. Uncropped blots showing all the bands with all molecular weight markers are shown in Supplementary Figure S3.

Next, to evaluate the levels of association of GC-AG Nef^mut^ with exosomes, HEK-293T cells were transfected with DNA vectors expressing either Nef^mut^/E7 or GC-AG Nef^mut^/E7. After 48 h, both cells and supernatants were harvested. Then, cell lysates and exosomes isolated from supernatants were analyzed by Western blot assay. Consistent with the previous analysis, the cell-associated steady-state levels of the two Nef^mut^-derivatives appeared similar. Conversely, and differently from the parental isoform, GC-AG Nef^mut^/E7 was no longer detectable in exosomes isolated from the cell supernatants (Figure 5). Thus, the amino acid substitutions at the N-terminus, besides influencing the intracellular localization, abolished the accumulation of GC-AG Nef^mut^/E7 into exosomes. We concluded that the DNA vector expressing GC-AG Nef^mut^/E7 represents a reagent suitable to establish the role of in vivo engineered EVs in the CD8^+^ T-cell immunity induced by DNA i.m. injection.

### 3.3. Nef^mut^-Mediated EV Engineering Is Necessary for the Induction of CD8^+^ T-Cell Immunity

**Figure 6:**
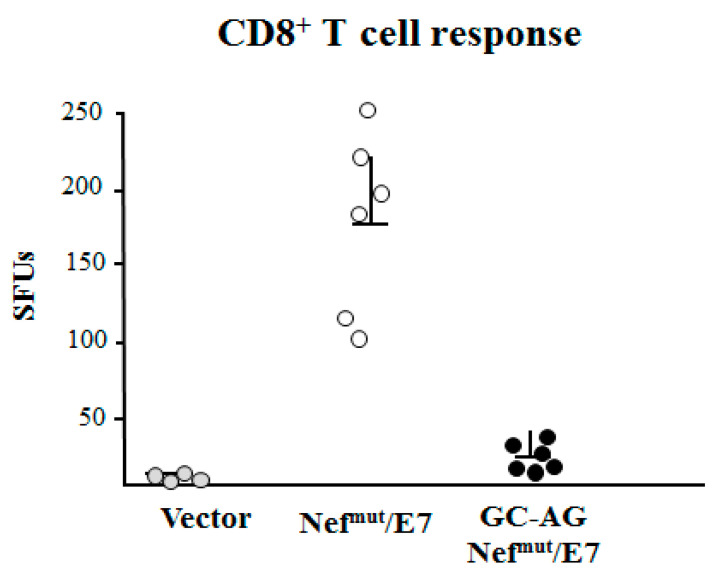
HPV16-E7-specific CD8^+^ T-cell immunity induced in mice after i.m. DNA injection. CD8^+^ T-cell immune response in C57 Bl/6 mice inoculated i.m. with the DNA vectors expressing either Nef^mut^/E7 or GC-AG Nef^mut^/E7 DNA vectors. As controls, mice were inoculated with void vector. At the time of sacrifice, 2.5 × 10^5^ splenocytes were incubated o.n. with or without 5 μg/mL of either unrelated or E7-specific peptides in triplicate IFN-γ ELISpot microwells. Shown are the numbers of IFN-γ spot-forming units (SFU)/well as mean values of triplicates after subtraction of mean values measured in wells of splenocytes treated with the unspecific peptide. Reported are cumulate data from two independent experiments. Intragroup mean values + standard deviations are also reported.

Next, we evaluated the immunogenicity of DNA vectors expressing Nef^mut^/E7 and its GC-AG mutant. C57 Bl/6 mice were inoculated i.m. in each quadriceps with 50 μg of the two DNA vectors and, as control, with equal amounts of void vector. The inoculations were repeated 14 days later, and after an additional 14 days, mice were sacrificed. Splenocytes were then isolated and cultured overnight in IFN-γ ELISpot microwells in the presence of either unrelated or E7-specific nonamers. We noticed a sustained E7-specific cell activation in splenocytes from mice inoculated with Nef^mut^/E7-expressing vector but not in those from mice injected with GC-AG Nef^mut^/E7 (Figure 6). Thus, the GC to AG amino acid substitutions at N-terminus of Nef^mut^/E7 strongly affected the downstream induction of antigen-specific CD8^+^ T-cell activity. Taken together, these data strongly enforce the idea that the in vivo generation of EVs uploading Nef^mut^-derivatives is the key event for the CD8^+^ T-cell immune response.

## 4. Discussion

The unique EV uploading efficiency of Nef^mut^ led us to envision a novel strategy based on engineered exosomes aimed at delivering antigens to antigen-presenting cells (APCs) [4]. When the method was translated in vivo, we noticed that the injection of Nef^mut^-based engineered exosomes induced an antigen-specific CD8^+^ T-cell immune response in the absence of antibody production [8]. As a further evolution of the system, immunogenic nanovesicles were generated in vivo by i.m. injection of Nef^mut^-based DNA vectors in a way that EVs constitutively released by muscle cells were engineered with Nef^mut^-derivatives [5,6,7]. This strategy has proven successful in terms of immunogenicity and therapeutic effects, leaving, however, still unresolved questions regarding the actual mechanism of action.

In particular, the main issue concerned the role of engineered EVs in the downstream induction of antigen-specific CD8^+^ T-cell response. Although they were detected in the plasma of injected mice, only suggestive but not conclusive experimental evidence about their actual immunogenicity was achieved. The GC-AG mutant was instrumental in addressing such a critical issue.

We observed that the GC-AG mutation at the Nef^mut^/E7 N-terminus abrogated the prevalent intracellular localization at the inner plasma membrane leaflet we previously observed in both human and mouse cells. On this subject, we [5,6,7] and others [17] reproducibly demonstrated that the properties of Nef and mutants thereof observed in mouse cells perfectly reproduced those detectable in human cells. Hence, we assume that the here-described in vitro evidence obtained with human cells was also relevant for the interpretation of data subsequently achieved in mice.

Nef^mut^/E7 and its mutant showed similar intracellular stability. This evidence gave significance to the comparison between the two isoforms in terms of both exosome uploading and immunogenicity in mouse since we can exclude that the results relied on different intracellular accumulation/degradation.

We observed that the immunogenicity was almost completely abrogated in mice i.m. injected with GC-AC Nef^mut^/E7 vector, which expresses a Nef^mut^-derivative unable to associate with exosomes. Hence, we concluded that the CD8^+^ T-cell immunity strictly depended on the production of in vivo engineered EVs. On the other hand, differences in Nef intracellular localization do not correlate with the downstream immunogenicity, as documented by the lack of immune response after i.m. injection of a vector expressing wtNef, i.e., a Nef isoform binding cell membrane similar to Nef^mut^ [6]. The low levels of anti-E7 CD8^+^ T immunity we detected in a number of mice injected with this vector may account for the already described often limited CD8^+^ T-cell immunity typically produced after i.m. injection of antigen-expressing DNA vectors [18,19,20,21]. This effect can be generated by cross-presentation of vector-expressed antigens after ingestion of dead muscle cells by APCs, and/or entry of DNA in resident APCs, and presentation of antigen-derived peptides in MHC Class I.

Simplicity, flexibility, and cost-effectiveness are some of the peculiarities of the Nef^mut^-based vaccine platform. In view of its originality and potential usefulness, investigating the underlying mechanism of action was mandatory. Here presented data represent the first step towards a clarification of the events occurring in vivo after i.m. injection of DNA vectors expressing Nef^mut^ derivatives. Uncovering still unclear mechanistic aspects will help to further improve the overall efficacy of the Nef^mut^-based vaccine platform, which would be of utility to fight both infectious and tumor diseases.

## Supplementary Materials

The following are available online at https://www.mdpi.com/2076-393X/8/2/243/s1, Figure S1: Uncropped blots showing all the bands with all molecular weight of Figure 3—Analysis of expression of GC-AG Nefmut/E7. Western blot analysis of total cell lysates from HEK-293T cells transfected with DNA vectors expressing the indicated Nefmut-based fusion products; Figure S2: Uncropped blots showing all the bands with all molecular weight markers of Figure 4—Analysis of stability of GC-AG Nefmut/E7. Comparative stability analysis between Nefmut/E7 and GC-AG Nefmut/E7 fusion proteins in total lysates from HEK-293T cells transfected with the respective DNA vectors; Figure S3: Uncropped blots showing all the bands with all molecular weight markers of Figure 5—Detection of Nefmut/E7 based fusion products in transfected cells and exosomes. Shown is the Western blot analysis of total lysates from the same number of HEK293T cells transfected with DNA vectors expressing either Nefmut/E7 or GC-AG Nefmut/E7 (left panels). Equal volumes of buffer, where purified exosomes were resuspended after differential centrifugations of the respective supernatants, were also analyzed (right panel).
