# Supplementary material for: N-Terminal Fatty Acids of NEFMUT Are Required for the CD8+ T-Cell Immunogenicity of In Vivo Engineered Extracellular Vesicles
Source: Vaccines (Basel). 2020 May 22;8(2):243. doi: 10.3390/vaccines8020243 (PMC7350016; doi:10.3390/vaccines8020243)
Supplement: Supplementary file 1 [file vaccines-08-00243-s001.zip › Figure S2.pdf]

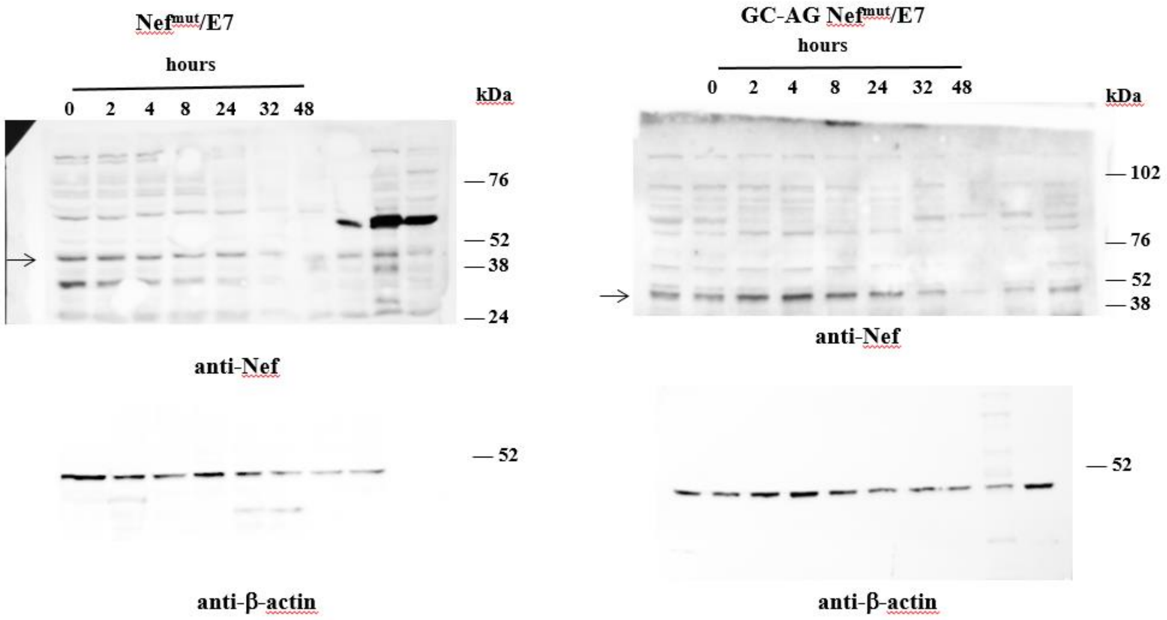

Figure S2. Uncropped blots showing all the bands with all molecular weight markers of Figure 4—Analysis of stability of GC-AG Nef^{mut}/E7. Comparative stability analysis between Nef^{mut}/E7 and GC-AG Nef^{mut}/E7 fusion proteins in total lysates from HEK-293T cells transfected with the respective DNA vectors.
