# Supplementary material for: N-Terminal Fatty Acids of NEFMUT Are Required for the CD8+ T-Cell Immunogenicity of In Vivo Engineered Extracellular Vesicles
Source: Vaccines (Basel). 2020 May 22;8(2):243. doi: 10.3390/vaccines8020243 (PMC7350016; doi:10.3390/vaccines8020243)
Supplement: Supplementary file 1 [file vaccines-08-00243-s001.zip › Figure S3.pdf]

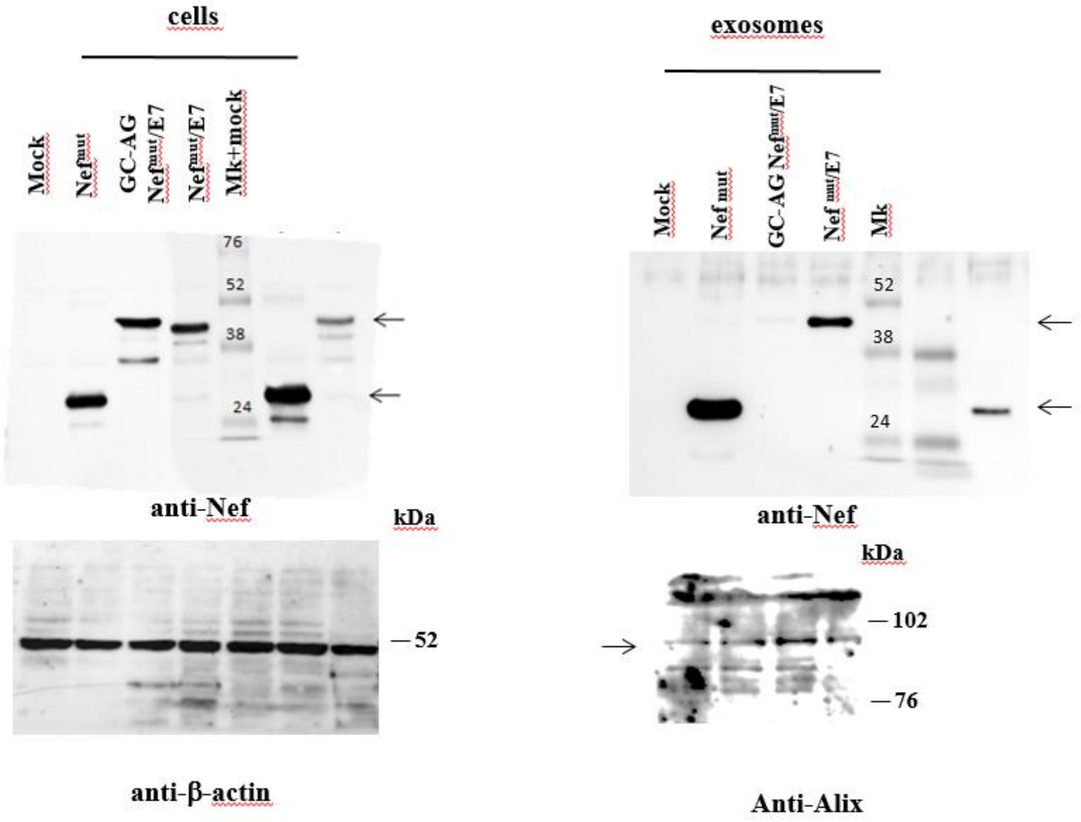

Figure S3. Uncropped blots showing all the bands with all molecular weight markers of Figure 5—Detection of Nef^{mut}/E7 based fusion products in transfected cells and exosomes. Shown is the Western blot analysis of total lysates from the same number of HEK293T cells transfected with DNA vectors expressing either Nef^{mut}/E7 or GC-AG Nef^{mut}/E7 (left panels). Equal volumes of buffer, where purified exosomes were resuspended after differential centrifugations of the respective supernatants, were also analyzed (right panel).
