# Supplementary material for: N-Terminal Fatty Acids of NEFMUT Are Required for the CD8+ T-Cell Immunogenicity of In Vivo Engineered Extracellular Vesicles
Source: Vaccines (Basel). 2020 May 22;8(2):243. doi: 10.3390/vaccines8020243 (PMC7350016; doi:10.3390/vaccines8020243)
Supplement: Supplementary file 1 [file vaccines-08-00243-s001.zip › Figure S1.pdf]

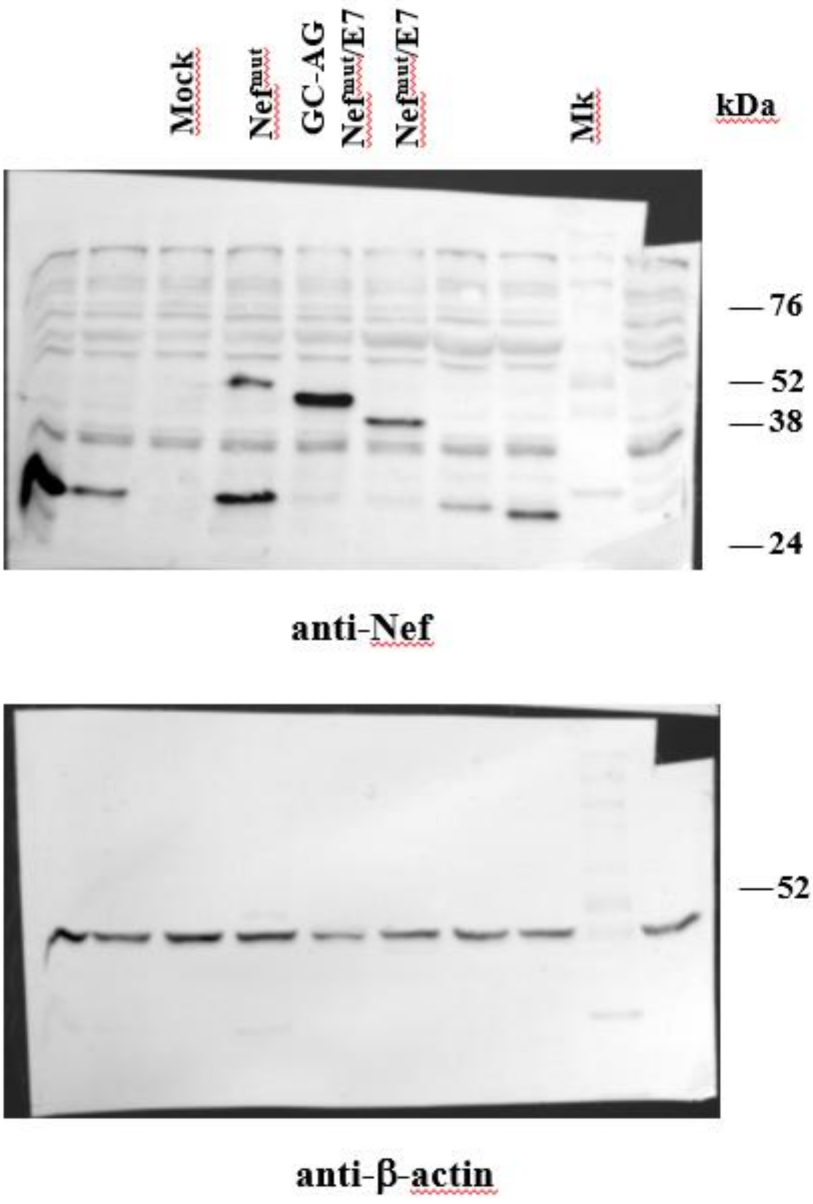


**Figure S1.** Uncropped blots showing all the bands with all molecular weight of Figure 3—Analysis of expression of GC-AG Nef$^{mut}$/E7. Western blot analysis of total cell lysates from HEK-293T cells transfected with DNA vectors expressing the indicated Nef$^{mut}$-based fusion products.
